# Use of a Safe, Reproducible, and Rapid Aerosol Delivery Method to Study Infection by *Burkholderia pseudomallei* and *Burkholderia mallei* in Mice

**DOI:** 10.1371/journal.pone.0076804

**Published:** 2013-10-02

**Authors:** Eric R. Lafontaine, Shawn M. Zimmerman, Teresa L. Shaffer, Frank Michel, Xiudan Gao, Robert J. Hogan

**Affiliations:** 1 Department of Infectious Diseases, University of Georgia, Athens, Georgia, United States of America; 2 Department of Microbiology, University of Georgia, Athens, Georgia, United States of America; 3 Department of Veterinary Biosciences and Diagnostic Imaging, University of Georgia, Athens, Georgia, United States of America; University of Toledo School of Medicine, United States of America

## Abstract

*Burkholderia pseudomallei*, the etiologic agent of melioidosis, is a saprophytic bacterium readily isolated from wet soils of countries bordering the equator. *Burkholderia mallei* is a host-adapted clone of *B. pseudomallei* that does not persist outside of its equine reservoir and causes the zoonosis glanders, which is endemic in Asia, Africa, the Middle East and South America. Infection by these organisms typically occurs via percutaneous inoculation or inhalation of aerosols, and the most common manifestation is severe pneumonia leading to fatal bacteremia. Glanders and melioidosis are difficult to diagnose and require prolonged antibiotic therapy with low success rates. There are no vaccines available to protect against either *Burkholderia* species, and there is concern regarding their use as biological warfare agents given that *B. mallei* has previously been utilized in this manner. Hence, experiments were performed to establish a mouse model of aerosol infection to study the organisms and develop countermeasures. Using a hand-held aerosolizer, BALB/c mice were inoculated intratracheally with strains *B. pseudomallei* 1026b and *B. mallei* ATCC23344 and growth of the agents in the lungs, as well as dissemination to the spleen, were examined. Mice infected with 10^2^, 10^3^ and 10^4^ organisms were unable to control growth of *B. mallei* in the lungs and bacteria rapidly disseminated to the spleen. Though similar results were observed in mice inoculated with 10^3^ and 10^4^ *B. pseudomallei* cells, animals infected with 10^2^ organisms controlled bacterial replication in the lungs, dissemination to the spleen, and the extent of bacteremia. Analysis of sera from mice surviving acute infection revealed that animals produced antibodies against antigens known to be targets of the immune response in humans. Taken together, these data show that small volume aerosol inoculation of mice results in acute disease, dose-dependent chronic infection, and immune responses that correlate with those seen in human infections.

## Introduction

*Burkholderia pseudomallei* is a Gram-negative bacterium found in water and wet soils of endemic areas bordering the equator, particularly Southeast Asia and Northern Australia [1-10]. The organism infects most mammals and causes the disease melioidosis in humans [1-10]. Clinical manifestations vary greatly and may present as flu-like symptoms, benign pneumonitis, acute/chronic pneumonia, or fulminating septicemia. Infection generally occurs via inhalation of aerosols or through skin abrasions, and the risk of contracting the disease is proportional to the concentration of *B. pseudomallei* in soil and water. In endemic areas, heavy rainfalls result in a shift from percutaneous inoculation to inhalation as the primary mode of infection, which also leads to a more severe illness. Melioidosis commonly affects the lungs and is characterized by the spread and seeding of bacteria to the spleen, liver, and lymph nodes. The incubation period is not clearly defined, but may range from 2 days to many years. Risk factors include diabetes mellitus, alcoholism, cirrhosis, thalassemia, and chronic lung disease. Most infected patients become bacteremic and the mortality rates remain high (19-51%) despite aggressive antimicrobial therapy. *Burkholderia pseudomallei* is refractory to most antibiotics and resistance mechanisms include efflux pumps and β-lactamases [1-10]. The recommended treatment for melioidosis entails the use of ceftazidime and meropenem (intensive phase) and TMP-SMX and co-amoxiclav (eradication phase) for several months [11]. Response to treatment is slow and eradication of *B. pseudomallei* is difficult to achieve, resulting in recrudescence [1-10]. Though considered exotic to the US and most European countries, *B. pseudomallei* is a leading cause of sepsis and bacteremic pneumonia in endemic areas, and melioidosis is recognized as an emerging infectious disease in many tropical regions of the world [12-14].

*Burkholderia mallei* is a non-motile, host-adapted clone of *B. pseudomallei* that does not persist outside of its equine reservoir and is endemic to parts of Asia, Africa, the middle East, and South America [8,9,15-21]. *Burkholderia mallei* causes the highly contagious zoonotic disease glanders, which primarily affects horses, mules, and donkeys. In humans, infection generally occurs by contact with infected animals via the cutaneous or respiratory route. Disease progression and pathology in humans and horses are similar, though the clinical presentation of any 2 cases in the same species, even if related by direct transmission, may vary. The clinical manifestations include febrile pneumonia with necrosis of the tracheobronchial tree, or pustular skin lesions and development of multiple abscesses (AKA farcy). Most patients become bacteremic and *B. mallei* disseminates to the liver, spleen, and lymph nodes where it rapidly causes necrosis. The course of disease may range from acute and rapidly fatal to very slow and protracted with alternating remissions and exacerbations. Even with antibiotic treatment, the mortality rate for human glanders is 50%. Like *B. pseudomallei*, *B. mallei* is resistant to most antibiotics [8,9,15-24].

There is concern that *B. mallei* and *B. pseudomallei* may be used as agents of biological warfare, especially since *B. mallei* has already been utilized in this manner [19,25-30]. *Burkholderia pseudomallei* was studied by the US and Russia as a potential bioweapon, but was never used in this capacity [25,31,32]. However, the ease of acquiring *B. pseudomallei* from the environment coupled with its resistance to antibiotics, severity of illness upon aerosol inoculation, difficulties in diagnosis, and persistence in the host make this possibility a serious concern. If the organism was cultured, concentrated, and delivered as an aerosol, significant casualties would ensue. For these reasons, the US Federal Select Agent Program classifies *B. pseudomallei* and *B. mallei* as Tier 1 agents, and the development of countermeasures is a priority. Protection against the pulmonary form of glanders and melioidosis is of particular interest as the lung is the most likely portal of entry for the organisms during a biologic attack.

The most commonly used surrogate to study *B. mallei* and *B. pseudomallei* is the mouse, and intranasal inoculation has generally been utilized to model the aerosol route of infection [8,33-40]. The method is quick, cost-effective, and relies on the mouse breathing in the agent, hence mimicking the natural route of aerosol infection for human disease. The availability of transgenic strains and the wealth of murine immunological tools provide a powerful platform to study and understand pathogenesis by the organisms and develop anti-infective approaches. However, unlike humans, mice are obligate nasal breathers and possess a greater surface area in their upper respiratory tract in addition to enhanced olfactory senses [41,42]. Consequently, intranasal inoculation results in a significant proportion of the inoculum remaining in the nasal passages (and not reaching the lungs) [43,44], and leads to high rates of infection of the nasal associated lymphoid tissues and central nervous system (via olfactory tissues), which are not common in humans [37,45,46]. Moreover, intranasal inoculation entails inhalation of a liquid suspension, not small-aerosolized particles. Whole-body and nose-only aerosol exposure models have been utilized to study *B. mallei* and *B. pseudomallei* [47-52]. Though these platforms deliver small aerosol particles, they too depend on the normal the mouse breathing in the agent through the nasal passageways and favor the aforementioned alternative portals of entry into the body. Furthermore, whole-body and nose-only exposure models require specialized equipment and dedicated BSL3 laboratory space, which can be cost-prohibitive and not practical at most research facilities.

The aim of this study was to develop a non-invasive, rapid, reproducible, practical, and safe aerosol delivery method to study the pulmonary form of glanders and melioidosis in BALB/c mice, without the potentially misleading effect of infection of the nasal associated lymphoid tissues and central nervous system. This model could then be used to investigate the pathogenesis of *B. pseudomallei* and *B. mallei* and aid in the development of relevant countermeasures for these highly pathogenic organisms.

## Materials and Methods

### Bacterial strains and growth conditions

*Escherichia coli* was cultured using Luria-Bertani (LB) medium (Fisher BioReagents) supplemented with 15 µg/mL chloramphenicol. *Burkholderia pseudomallei* strain 1026b [53] was grown on Tryptic Soy Agar (TSA; BD Difco™) for 18 hours at 37°C prior to infection. Blood, bronchoalveolar lavages, and tissue homogenates from mice infected with *B. pseudomallei* 1026b were plated on TSA containing 100 µg/mL polymyxin B and agar plates were incubated at 37°C for 48 hours in order to count colonies and calculate bacterial loads in tissues. *Burkholderia mallei* strain ATCC23344 [54] was routinely cultured on Brucella broth Agar supplemented with 4% (vol/vol) Glycerol (BAG, BD-BBL™). To prepare the inoculum for infection, *B. mallei* ATCC23344 was grown on BAG for 42 hours at 37°C. Homogenized tissues, lavage fluids, and blood from infected mice were spread onto BAG supplemented with 8 µg/mL polymixin B and incubated at 37°C for 72 hours to determine the number of viable *B. mallei* bacteria in tissues. All experiments with live *B. pseudomallei* and *B. mallei* were performed inside a Class II Biosafety Cabinet in a BSL3 laboratory and in compliance with the rules and regulations of the U.S. Federal Select Agent Program.

### Experimental animals and aerosol infection procedures

Female BALB/c mice (6-8 weeks of age) were purchased from Frederick National Laboratory for Cancer Research. Before inoculation, mice were anesthetized by injecting 2, 2, 2 Tribromoethanol (Sigma-Aldrich®) intraperitoneally at a dose of 250 mg/kg. Once anesthetized, mice were placed on their back in a specially designed workstand inclined at an angle of 45° (Hallowell EMC). This workstand is outfitted with a nylon wire, to suspend animals by their upper teeth, and lateral barriers to minimize movement during inoculation. Next, a fiber optic arm equipped with a light source (Fisher Scientific) was placed over the front of the throat of suspended animals in order to visualize the tracheal opening. The mouth of the mouse was opened and the tongue was moved to the side with forceps. A modified pediatric otoscope (Braintree Scientific, Inc) was then used to guide the blunt needle portion of a MicroSprayer® model I-1C (PennCentury™) inside the tracheal opening and between the vocal cords. A total of 50 µL of bacterial suspension was subsequently delivered into the lungs using the gas-tight, high-pressure syringe component of the MicroSprayer® device.

*Burkholderia pseudomallei* and *B. mallei* bacteria used to inoculate mice were cultured on agar plates and suspended in Phosphate-Buffered Saline (PBS) to an optical density of 250 Klett units using a Klett™ Colorimeter (Scienceware®), which corresponds to ~1X10^9^ colony forming units (CFU) per mL. The bacterial suspensions were serially diluted and 100 µL aliquots were immediately spread onto agar plates to determine the number of CFU present in the inoculum. This back-titration of the inoculum was performed for all challenge experiments. Infected mice were monitored twice daily for clinical signs of illness over a period of up to 30 days. At the indicated experimental end points, animals were anesthetized and euthanized. Tissues (blood, lungs, spleen) were aseptically collected, homogenized with disposable tissue grinders (Fisherbrand®), serially diluted, and plated on agar medium to calculate bacterial loads. Survival data was analyzed using the Kaplan-Meier method. LD_50_ values were calculated according to Reed and Muench [55].

Infected animals were monitored twice daily. Humane end-points were strictly observed. Mice exhibiting signs of moderate to severe discomfort were euthanized. This was accomplished by anesthetizing the animals with 2, 2, 2 Tribromoethanol followed by cervical dislocation. This procedure is in accordance with the AVMA Guidelines on Euthanasia. The following grading system was used to determine if euthanasia was appropriate: WEIGHT LOSS >20% = 3 ***points***; BODY CONDITION (rough coat) and EMACIATION (evident segmentation of vertebral column and/or dorsal pelvic bones readily palpable) = 1 ***point***; MODERATE DYSPNEA (based on thoracic movement and respiratory rate) = 1 ***point***; HUNCH BACK = 1 ***point***; FAILURE TO RESPOND TO STIMULI = 3 ***points***; CONJUNCTIVITIS (eyes swelling shut, no apparent ophthalmic discharge) = 1 ***point***. Any animal with a point score ≥3 was humanely euthanized. Pain and suffering of the animals were minimized by performing all procedures under anesthesia and by observing the aforementioned humane end-points. Food and water were provided *ad libitum*. Analgesics were not used as they may have affected the experimental outcomes of the studies.

To investigate pathologic changes induced by the organisms in the lung, bronchoalveolar lavage cells were collected and inspected by microscopy. Under anesthesia, the trachea of mice was exposed and an incision was made through the midline. The blunt end of a 23-gauge needle was next inserted through the tracheal incision. Using a 1-cc syringe, sterile 0.9% saline was infused into the lungs and fluids (~ 1-ml) were recovered by gentle suction. These bronchoalveolar lavage fluids (BALF) were processed within 1-2 hr of collection to insure cell morphology was maintained. Specifically, 0.3-mL aliquots were placed into wells of duplicate disposable sample concentrators, each of which containing a slide. The BALF were then centrifuged at 1,000-rpm for 5-min using a StatSpin® Cytofuge (Iris® Sample Processing). The slides were removed from the cell concentrators, air-dried for 10-min, fixed by immersing in 100% anhydrous methanol, and stained with modified Wright-Giemsa using an automated Aerospray® Hematology Slide Stainer (Wescor® Biomedical Systems). All samples were examined by a board-eligible veterinary clinical pathologist. Cytologic evaluations were performed by microscopy using an Olympus Bx51 microscope (Olympus Corporation). Images were captured using an Olympus DP71 camera and software (Olympus Corporation).

### Recombinant DNA methods, PCR, and cloning

Standard molecular biology techniques were performed as described elsewhere [56,57]. Genomic DNA was obtained from plate-grown bacteria using the Easy-DNA™ kit (Invitrogen™ Life Technologies™). Platinum *Pfx* DNA Polymerase was used in cloning experiments per the manufacturer’s recommendations (Invitrogen™ Life Technologies™). A 2.2-kb amplicon encompassing amino acids (aa) 25-750 of the *B. pseudomallei* 1026b BoaA protein (GenBank accession number EF423807) was generated with primers P1 (5’- CGC CAC GTG AAT GGG ACC GTC AAC TCG -3’; PmlI site underlined) and P2 (5’- GGT TAA TTA AAG ATT AGT GAT CTT CAC GGG -3’; PacI site underlined). This DNA fragment was excised from an agarose gel, purified with the High Pure PCR Product Purification Kit (Roche Applied Science), restricted with the endonucleases PmlI and PacI (New England Biolabs® Inc.), and ligated into the PmlI and PacI sites of the vector pETcoco-1 (specifies N-terminal His-tag, EMD Millipore), yielding plasmid pELHisBoaA. This plasmid was sequenced to verify that no mutations were introduced during PCR and to confirm that the protein expressed from pELHisBoaA corresponds to residue 25-750 of *B. pseudomallei* 1026b BoaA joined to six N-terminal histidine residues. Plasmid DNA used as template in sequencing reactions was obtained with the QIAprep Spin Miniprep Kit (Qiagen). A similar approach was used to obtain the plasmid pELHisBPSL1631-BMA1027 (expresses residues 392-1068 of *B. pseudomallei* 1026b BPSL1631-BMA1027 ORF), and pELHisBPSS0908-BMAA1324 (specifies residues 25-690 of *B. pseudomallei* 1026b BPSS0908-BMAA1324 ORF). The PCR products cloned into pELHisBPSL1631-BMA1027, and pELHisBPSS0908-BMAA1324 were amplified with primers P3 (5’- CCC AAG CTT CAG CTT TAC ACG CTC CAG -3’; HindIII site underlined) and P4 (5’- GGT TAAT TAA AGC AAC TGG CCG ACG TTG AC -3’; PacI site underlined), and P5 (5’- CCC AAG CTT GGC GAG AAC GCC TAT GCC GGC -3’; HindIII site underlined) and P6 (5’- GGT TAA TTA AAG GAC CTT CTG ATC CGT GTA CTG -3’; PacI site underlined), respectively. Genomic DNA was used as the template in all PCR-based cloning experiments. The designation BPSxxxxx-BMAxxxxx refers to the locus tag numbers of the ORF under study in the annotated genomic sequence of *B. pseudomallei* strain K96243 [58] and *B. mallei* ATCC23344 [54], respectively, which are readily available through NCBI.

### Nucleotide sequence analysis

Plasmids were sequenced at the University of Michigan sequencing core (http://seqcore.brcf.med.umich.edu/). Chromatograms were analyzed and assembled with the Sequencher software (Gene Codes Corporation). Sequence analysis was performed using Vector NTI (Invitrogen™ Life Technologies™).

### Purification of selected antigens

His-tagged recombinant proteins were obtained as previously outlined by our laboratory [59,60]. Briefly, the plasmids pELHisBoaA, pELHisBPSL1631-BMA1027, and pELHisBPSS0908-BMAA1324 were introduced in the *E. coli* strain TUNER™ (EMD Millipore) for the purpose of overexpressing and purifying recombinant proteins. Expression was induced by adding isopropyl-β-d-thiogalactopyranoside (IPTG, final concentration of 1 mM) to broth cultures and incubating for 5 hours at 37°C with agitation (200-rpm). Bacteria were pelleted, followed by treatment with the BugBuster® HT protein extraction reagent (EMD Millipore) supplemented with rLysozyme™ (EMD Millipore) under the recommended conditions. Recombinant proteins were then purified using the His Bind Resin® System (EMD Millipore) per the manufacturer’s instructions. Protein concentrations were determined with a bicinchoninic acid (BCA) Protein assay kit (Thermo Scientific Pierce). Donald E. Woods at the University of Calgary kindly provided capsular polysaccharides (CPS) and oligosaccharide chain of LPS (OPS). The molecules were purified from the *B. pseudomallei* strains SR1015 (CPS^-^ mutant of *B. pseudomallei* 1026b [61], used to purify OPS) and MB100 (LPS^-^ mutant of *B. pseudomallei* 1026b [62], used to purify CPS).

### ELISA

Duplicate wells of Immulon™ 2HB plates (Thermo Scientific Nunc) were coated overnight at 4°C with ~1 µg of antigen. Excess unbound antigen was removed by washing the wells with PBS+0.05% Tween 20, and the wells were then filled with PBS+0.05% Tween 20 containing 3% (wt/vol) dry milk and incubated for 1 hour at room temperature. After washing with PBS+0.05% Tween 20, the wells were probed overnight at 4°C with primary antibodies diluted in PBS+0.05% Tween 20 + 3% dry milk. After this incubation, the wells were washed with PBS+0.05% Tween 20, followed by overnight incubation at 4°C with secondary antibodies conjugated to HRP (SouthernBiotech) and diluted in PBS+0.05% Tween 20+3% dry milk. After washing off the excess secondary antibodies with PBS+0.05% Tween 20, 100 µL of the SureBlue™ TMB Microwell Peroxidase Substrate (KPL) was added to wells. Color development, indicative of antibody binding to antigen, was measured by determining the absorbance of well contents at a wavelength of 650 nm using a μQuant™ Microplate Spectrophotometer (BioTek®).

### Animal research ethic statement

This study was carried out in strict accordance with the recommendations in the Guide for the Care and Use of Laboratory Animals of the National Institutes of Health. The protocol was approved by the University of Georgia’s Institutional Animal Care and Use Committee as well as by the Institutional Biosafety Committee. All efforts were made to minimize animal suffering.

### Statistics

Statistical analyses were performed using Prism 6.0 (GraphPad Software, Inc.).

## Results and Discussion

### Murine model of aerosol infection

*Burkholderia mallei* and *B. pseudomallei* infections generally occur via percutaneous inoculation or inhaling aerosols. The respiratory route of infection, and ensuing pulmonary disease, are also of particular concern with respect to the use of *B. mallei* and *B. pseudomallei* as agents of biological warfare. Hence, we developed a mouse model of aerosol inoculation to study the organisms. Our model entails the use of a MicroSprayer® (PennCentury™) to deliver bacteria directly into the lungs. The device generates aerosols from the tip of a bent needle attached to a high-pressure stainless steel syringe that contains the agent. A modified pediatric otoscope is used to introduce the needle part of the MicroSprayer® into the trachea of anesthetized mice, and 50 µL of bacterial suspension is aerosolized into the airway. To our knowledge, this inoculation method has not been reported for *B. pseudomallei* or *B. mallei*.

**Figure 1:**
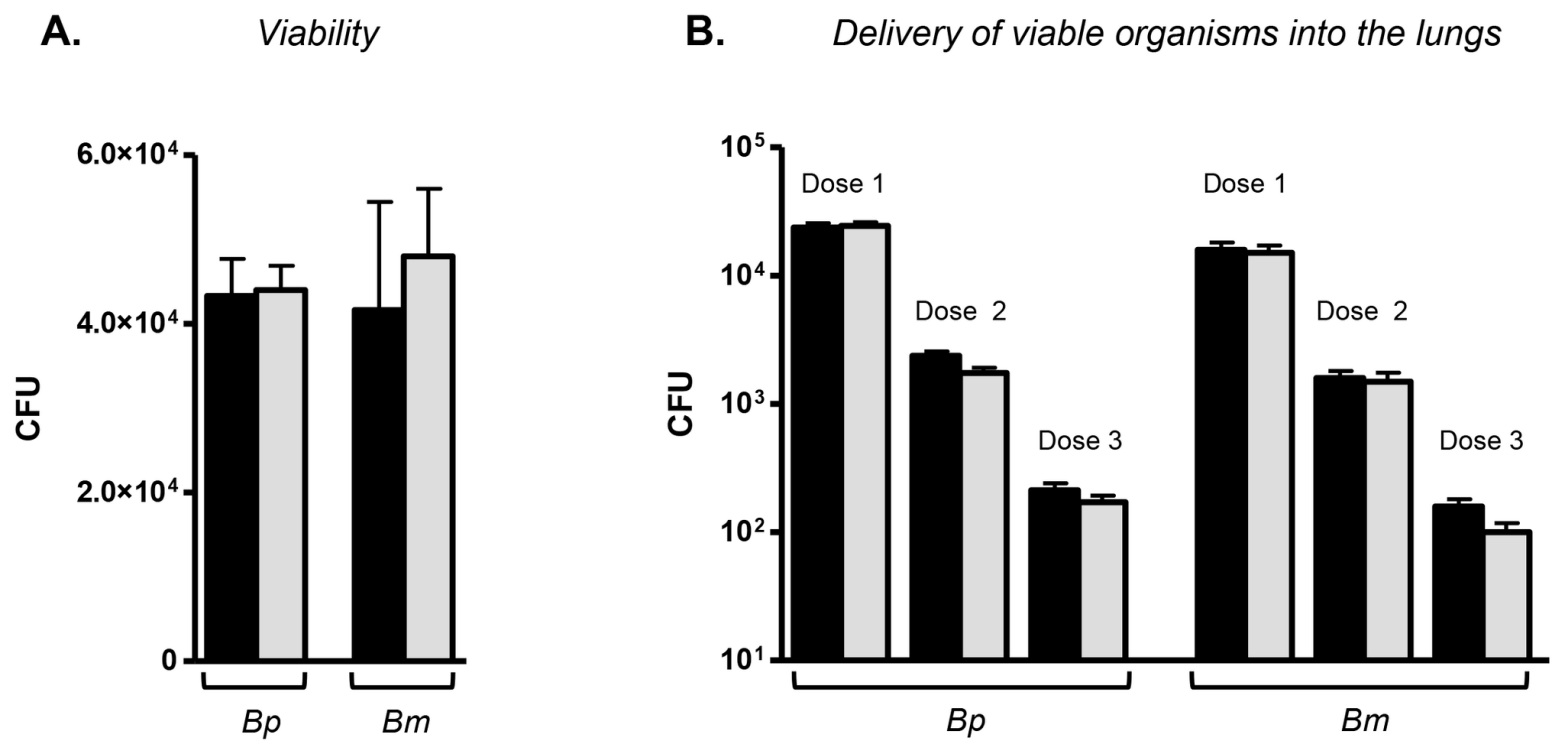
Viability of *B. pseudomallei* (*Bp*) and *B. mallei* (*Bm*) upon use of the MicroSprayer® and delivery of viable organisms into the murine lungs. Panel A: Bacteria were suspended in PBS to an optical density of 1X10^6^ bacteria/mL, serially diluted, and plated onto agar medium to calculate the number of viable organisms in 50 µL (black bars). The MicroSprayer® was then used to deliver 50 µL of bacterial suspensions into 1 mL of sterile PBS, which was serially diluted and plated onto agar medium to determine the number of viable bacteria (grey bars). Results are expressed as the mean (± standard error) colony forming units (CFU). These experiments were performed in triplicate on 2 separate occasions. Panel B: Bacteria were suspended in PBS to optical densities of 1X10^6^ (Dose 1), 1X10^5^ (Dose 2), and 1X10^4^ (Dose 3) bacteria/mL, serially diluted, and plated onto agar medium to calculate the number of viable organisms in 50 µL (black bars). The MicroSprayer® was then used to deliver 50 µL of bacterial suspensions into the lungs of mice (n=3 per dose). Thirty minutes post-inoculation, the mice were euthanized and their lungs were collected, homogenized, diluted, and plated onto agar medium to determine bacterial loads (grey bars). Results are expressed as the mean (± standard error) CFU. These experiments were performed on at least 2 separate occasions. The Mann-Whitney test was used to compare the number of viable organisms in 50 µL of bacterial suspension (i.e. before using the MicroSprayer®; black bars) to that in 1 mL PBS (panel A) or lung homogenates (panel B) after the use of the MicroSprayer® (i.e. grey bars). No statistically significant differences were noted.

The MicroSprayer® produces a mist of particles with a mean mass aerodynamic diameter of 8 µM [63]. Because the aerosol is generated by pushing fluid through an atomizer (sapphire) located at the tip of the needle part of the device, we tested whether this procedure affects the viability of *B. pseudomallei* and/or *B. mallei*. Bacterial suspensions containing 10^5^ organisms per mL were loaded into the high-pressure syringe component of the MicroSprayer® and a 50 µL dose was sprayed into 1 mL of PBS, serially diluted, and plated onto agar medium to calculate the number of viable bacteria. The results of these experiments are shown in Figure 1A and demonstrate that the use of the MicroSprayer® does not adversely impact viability of the agents. Experiments were also performed to verify that the number of viable organisms delivered into the lungs of mice is consistent with the number of bacteria in the inoculum. To accomplish this, plate-grown *Burkholderia* were suspended in PBS to concentrations of 1X10^6^, 1X10^5^ and 1X10^4^ cells per mL, serially-diluted, and plated onto agar medium to calculate the number of viable organisms in the inoculum prior to infection. The MicroSprayer® was then used to deliver the agents in the murine lungs. Thirty minutes post-inoculation, mice were euthanized (n=3 mice per dose) and their lungs were collected, homogenized, diluted, and plated on agar medium to determine bacterial loads. The results of these experiments are shown in Figure 1B and indicate that the number of organisms deposited into the lungs is equivalent to the inoculum.

**Figure 2:**
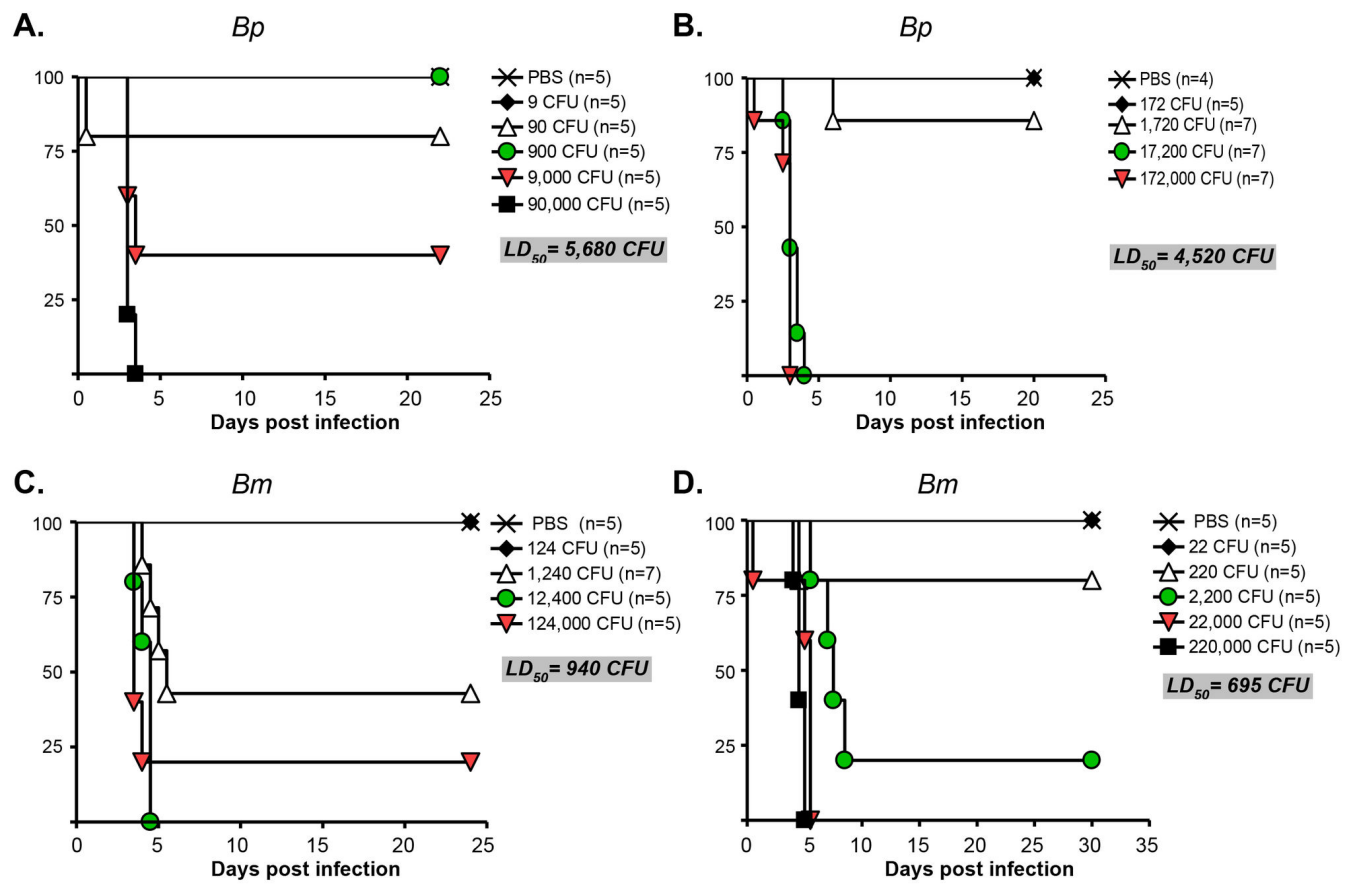
LD_50_ of *B. pseudomallei* 1026b (*Bp*) and *B. mallei* ATCC23344 (*Bm*) after inoculation with the MicroSprayer®. The MicroSprayer® was used to deliver the indicated number of bacterial CFU into the lungs of BALB/c mice. Animals were then monitored for clinical signs of illness and morbidity. Survival data were analyzed with the Kaplan-Meier method and the LD_50_ values were calculated according to Reed and Muench [55]. The number of animals/group is shown in parentheses. Control mice were inoculated with 50 µL of PBS using the MicroSprayer®. Panels A and B show 2 separate experiments to determine the LD_50_ of *B. pseudomallei* strain 1026b. Panels C and D show 2 independent experiments to determine the LD_50_ of *B. mallei* ATCC23344. With the exception of the survival curves for PBS and the lowest inoculating CFU dose, survival curves were found to be statistically different using the Logrank test for trend (panels A through D, p ≤ 0.05).

Next, we determined the median lethal dose of *B. pseudomallei* 1026b and *B. mallei* ATCC23344 according to the method of Reed and Muench [55]. Cohorts of mice were inoculated with 10-fold serial dilutions of the organisms using the MicroSprayer® and monitored for clinical signs of illness and morbidity for a period of up to 30 days. Based on the data shown in Figure 2, the calculated LD_50_ for the agents are 5,100 ± 820 (*B. pseudomallei* 1026b) and 818 ± 173 CFU (*B. mallei* ATCC23344). The value for strain ATCC23344 is consistent with published reports utilizing alternative aerosol delivery methods. Using the intranasal route of inoculation, the LD_50_ has been reported to be 820 CFU [39]. The use of whole body aerosol exposure chamber produced values of 1X10^3^-1.8X10^3^ organisms [47,50,64]. The LD_50_ of *B. pseudomallei* 1026b in our model is higher than that reported for the intranasal route of inoculation and whole body exposure chamber, which were calculated to be 1 X 10^3^ and 10 CFU, respectively [39,50]. Factors such as the age and weight of mice, and how bacteria were cultured prior to infection (i.e. broth versus plate-grown), may account for this difference. The intranasal route of inoculation and the use of the whole body exposure chamber entail mice breathing in the agent through the nasal passages and can cause infection of the nasal associated lymphoid tissues and central nervous system (in addition to pulmonary disease), which could have contributed to the lower LD_50_ in these models. Intratracheal inoculation with the MicroSprayer® circumvents the mouse nasal passageways and may have reduced the potentially confounding complications due to infection of the nasal associated lymphoid tissues and central nervous system, resulting in a higher LD_50_ value for *B. pseudomallei* 1026b.

**Figure 3:**
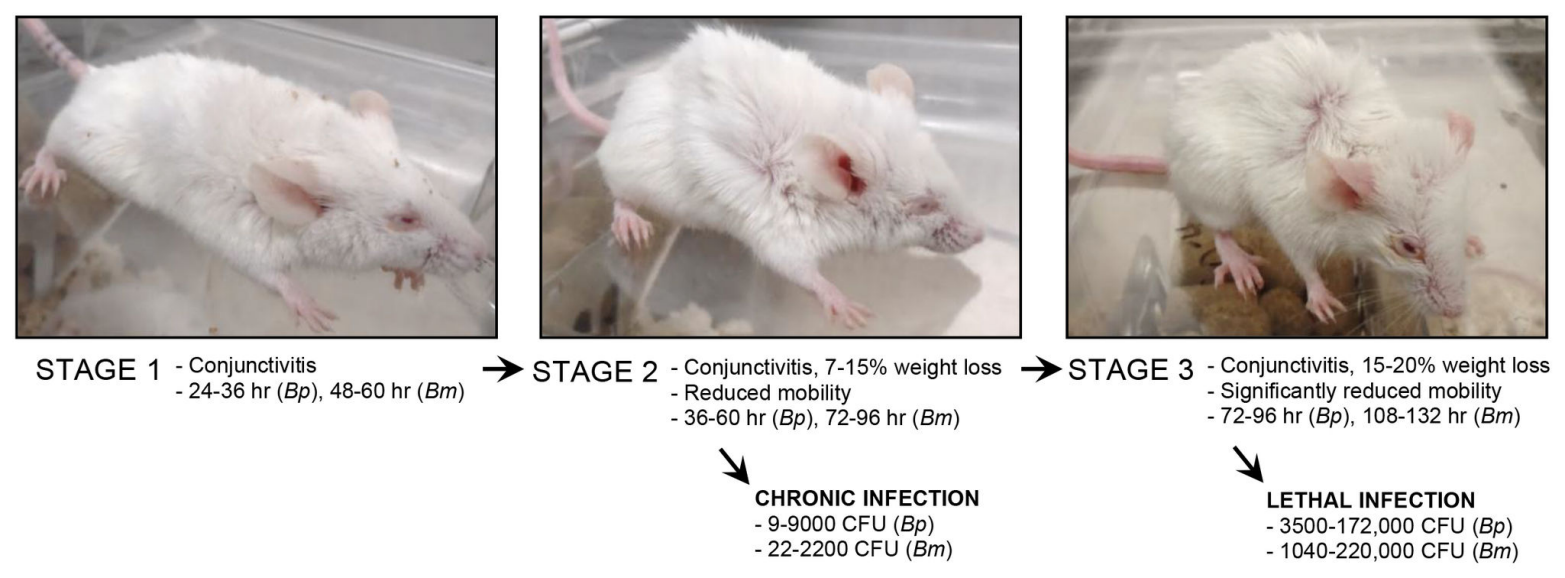
Clinical progression of disease after inoculation with the MicroSprayer®.

The clinical progression of disease was found to be very similar for both organisms and can be divided in the 3 stages depicted in Figure 3. Mice in Stage 2 develop chronic infection (characterized by lung granulomas and spleen abscesses, data not shown) or progress to Stage 3, which is characterized by conjunctivitis, significantly reduced mobility, considerable weight loss, and difficulty breathing. Animals that progress to Stage 3 invariably succumbed to infection within 48 hours. All animals infected with the MicroSprayer® developed disease, even with inoculating doses as low as 9 (*B. pseudomallei* 1026b, Figure 2A) and 22 (*B. mallei* ATCC23344, Figure 2D) organisms. Taken together, these data indicate that we have developed a highly reproducible, sensitive, consistent, and accurate aerosol inoculation method to study *B. mallei* and *B. pseudomallei* infection.

### Replication and dissemination of agents after aerosol inoculation

To examine the kinetics of replication and dissemination, mice were challenged with 10^2^, 10^3^ and 10^4^ bacteria, which correspond to roughly 0.1, 1 and 10 LD_50_. Animals were then sacrificed at four different time points post-challenge (24, 48, 72 and 96-hr), and bacterial loads in the lungs, spleen and blood were determined. Additionally, lungs were collected 30-min after inoculation to calculate the number of viable organisms administered.

**Figure 4:**
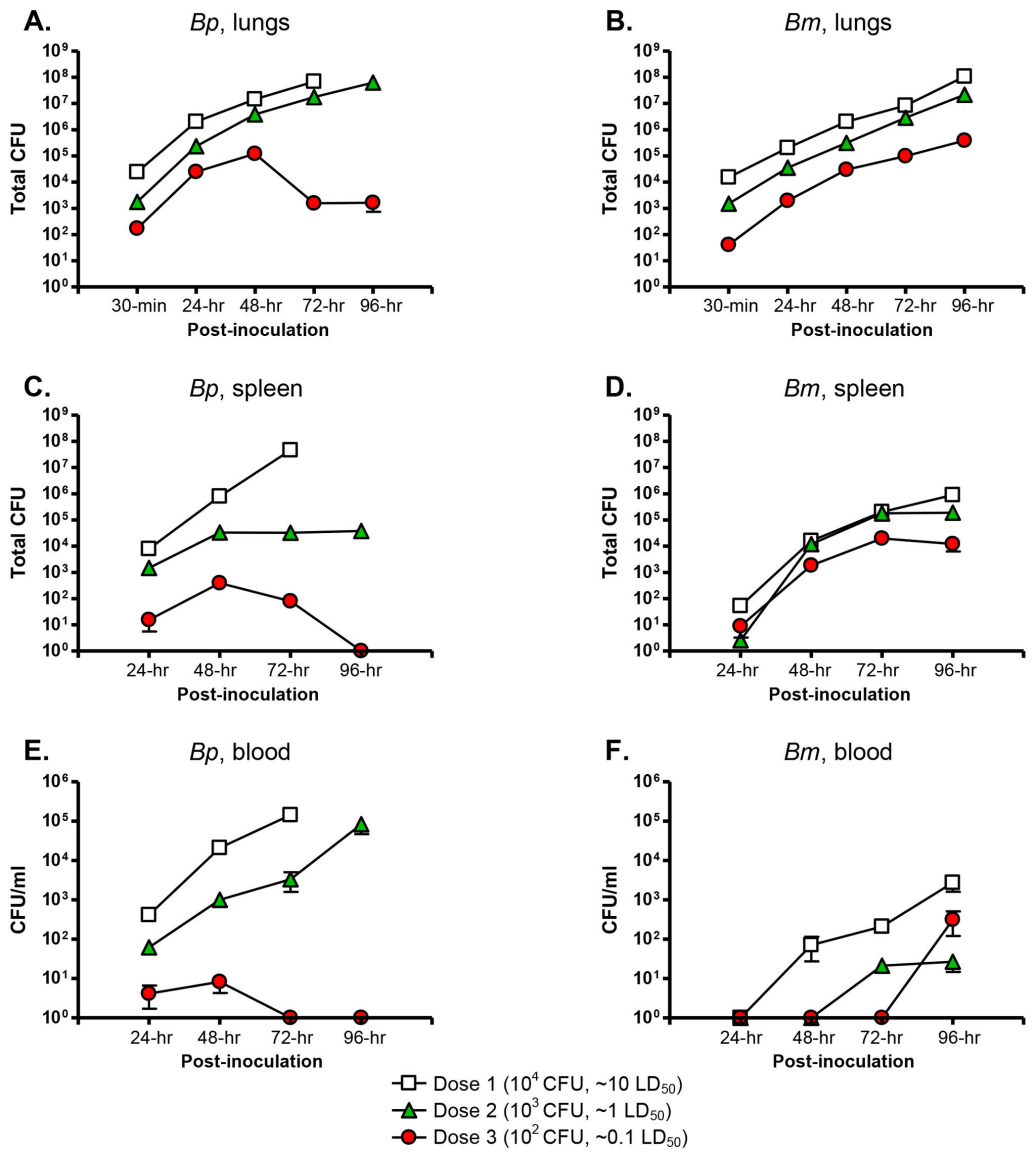
Bacterial loads in lungs, spleen and blood after inoculation with the MicroSprayer®. Bacteria were suspended in PBS to optical densities of 1X10^6^ (Dose 1), 1X10^5^ (Dose 2), and 1X10^4^ (Dose 3) bacteria/mL, serially diluted, and plated onto agar medium to calculate the number of viable organisms in 50 µL. The MicroSprayer® was then used to deliver 50 µL of bacterial suspensions into the lungs of mice (n=15 per dose). At the indicated time points post-inoculation, mice (n=3 per dose) were euthanized and tissues were collected, homogenized, diluted, and plated onto agar medium to determine bacterial loads. Results are expressed as the mean (± standard error) total CFU/per organ (panels A, B, C, D) and mean (± standard error) CFU/ml of blood (panels E and F). These experiments were performed on at least 2 separate occasions. *Bp*= *B. pseudomallei* 1026b, *Bm*=*B. mallei* ATCC23344. Both organisms were first detected in the spleen at 24-hr post-challenge (panels C and D). *Bp* disseminated to the organ in significantly greater numbers than *Bm* at the challenge doses of 10 and 1 LD_50_ (Wilcoxon Signed Rank test p<0.0001).

The organ most heavily infected by *B. pseudomallei* and *B. mallei* was the lung (Figure 4A and 4B). Between 30-min and 48-hr post-infection, the number of viable *B. mallei* bacteria increased by 2 orders of magnitude, regardless of the dose used to inoculate animals (Figure 4B). Thereafter, bacterial numbers continued to increase, albeit at a slower rate, to reach a maximum of 10^8^ (dose 1, 10 LD_50_), 10^7^ (dose 2, 1 LD_50_), and 10^5^ (dose 3, 0.1 LD_50_) CFU at 96-hr. *Burkholderia pseudomallei* replicated at a faster rate than *B. mallei* during the first 48-hr of infection, as bacterial numbers increased 1000-fold for all 3 inoculating doses (Figure 4A). In mice infected with 10 LD_50_, *B. pseudomallei* loads reached ~ 5X10^7^ CFU by 72-hr, and all animals succumbed to infection before the 96-hr time point. In mice challenged with 1 LD_50_, the bacterial numbers rose from ~2X10^6^ to nearly 10^8^ organisms between 48 and 96-hr post-infection. In contrast, a 100-fold reduction in CFU was measured during the same time period in mice inoculated with the equivalent of 0.1 LD_50_ of *B. pseudomallei*.

Both agents were first detected in the spleen at 24-hr post-challenge (Figure 4C and 4D). *Burkholderia pseudomallei* disseminated to the organ in greater numbers than *B. mallei*, especially at the challenge doses of 10 and 1 LD_50_. Between 24 and 72-hr, the number of viable *B. mallei* bacteria increased by 4 orders of magnitude for all 3 inocula (Figure 4D). Thereafter, bacterial loads in animals infected with 0.1 and 1 LD_50_ reached a plateau of 10^5^ and 10^4^ CFU, respectively, whereas the number of *B. mallei* cells in the spleen of mice challenged with 10 LD_50_ continued to increase up to 10^6^ organisms at 96-hr. Twenty-four hours after infection, the spleen of mice inoculated with 10 LD_50_ of *B. pseudomallei* were heavily colonized and the agent continued to proliferate, nearly reaching 10^8^ bacteria at 72-hr. Between 24 and 48-hr post-challenge, the bacterial loads in the spleen of animals inoculated with 1 and 0.1 LD_50_ increased 50-fold. From that point forward, the bacterial counts remained constant in mice infected with 1 LD_50_, but steadily declined to undetectable levels by 96-hr in animals challenged with 0.1 LD_50_.

*Burkholderia pseudomallei* was first cultured from the bloodstream 24-hr post-infection and the bacterial counts were proportional to the number of organisms delivered in the lungs with the MicroSprayer® (Figure 4E). The presence of the agent in peripheral blood at 24-hr, and colonization of the spleen with commensurate bacterial numbers at the same time point (Figure 4C), suggest dissemination via the bloodstream. In mice infected with 1 and 10 LD_50_, the numbers of viable bacteria in blood continue to rise and reached 10^5^ CFU/ml at the experimental end-points (Figure 4E). Though *B. pseudomallei* was cultured from the bloodstream of animals infected with 0.1 LD_50_ during the first 48-hr post-challenge, the bacterial loads peaked at 10^1^ CFU/ml at 48-hr and no bacteria were cultured from peripheral blood thereafter. *Burkholderia mallei* was not recovered from the blood at 24-hr post-infection, regardless of the dose used to inoculate animals (Figure 4F). The organism was first detected 48-hr post-infection in mice challenged with 10 LD_50_, and only at the 72 and 96-hr time points in animals inoculated with 1 and 0.1 LD_50_, respectively. The early, rapid replication of *B. mallei* in the lungs, the colonization of the spleen at 24 and 48-hr (Figure 4D), and the absence of bacteria in the bloodstream at those specific time points (Figure 4F), especially in mice infected with 1 and 0.1 LD_50_, suggest that *B. mallei* was phagocytized by alveolar macrophages and disseminated primarily via the lymphatic system. This hypothesis is supported by the presence of phagocytosed bacilli in macrophages from bronchoalveolar lavages (see below). More definitive experiments are needed to determine the precise mechanism(s) by which the bacteria disseminate from the site of infection to peripheral sites.

Altogether, these experiments demonstrate that *B. pseudomallei* and *B. mallei* rapidly replicate to large numbers in the lungs and disseminate to colonize the spleen within 24-hr post-infection. At the lower challenge dose of 10^2^ organisms, replication of *B. pseudomallei* 1026b was controlled in the lungs, spleen and blood during the first 96-hr post infection, which correlated with mice developing chronic infection. This was not observed in animals infected with *B. mallei* ATCC23344, even though most mice infected with 10^2^ bacteria also developed chronic infection.

### Analysis of bronchoalveolar lavages collected from infected mice

**Figure 5:**
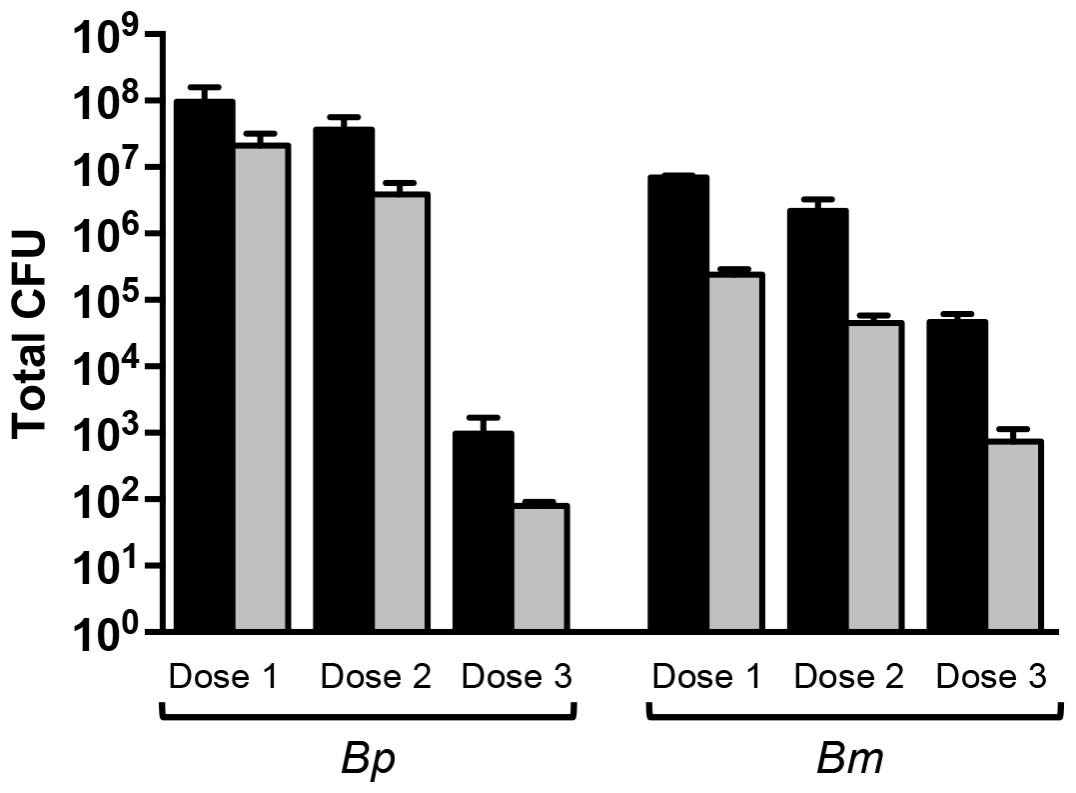
Bacterial loads in bronchoalveolar lavage fluids (BALF) and lungs after inoculation with the MicroSprayer®. Bacteria were suspended in PBS to optical densities of 1X10^6^ (Dose 1), 1X10^5^ (Dose 2), and 1X10^4^ (Dose 3) bacteria/mL, serially diluted, and plated onto agar medium to calculate the number of viable organisms in 50 µL. The MicroSprayer® was then used to deliver 50 µL of bacterial suspensions into the lungs of mice (n=3 per dose). Control mice were inoculated with 50 µL of sterile PBS. Seventy-two hours post-inoculation, the animals were euthanized and tissues (BALF, lungs) were collected, homogenized, diluted, and plated onto agar medium to determine bacterial loads. Results are expressed as the mean (± standard error) total CFU/per tissue. *Bp*= *B. pseudomallei* 1026b, *Bm*=*B. mallei* ATCC23344. Black bars= lung homogenates, grey bars=BALF.

To investigate pathologic changes induced by the organisms, bronchoalveolar lavage fluids (BALF) were collected from mice 72-hr post-challenge and examined by microscopy. Portions of these fluids (and lung homogenates) were also diluted and plated onto agar medium to determine bacterial loads (Figure 5). The animals were infected with 10^2^, 10^3^ and 10^4^ bacteria, while control mice were inoculated with 50 µL of sterile PBS using the MicroSprayer®.

**Figure 6:**
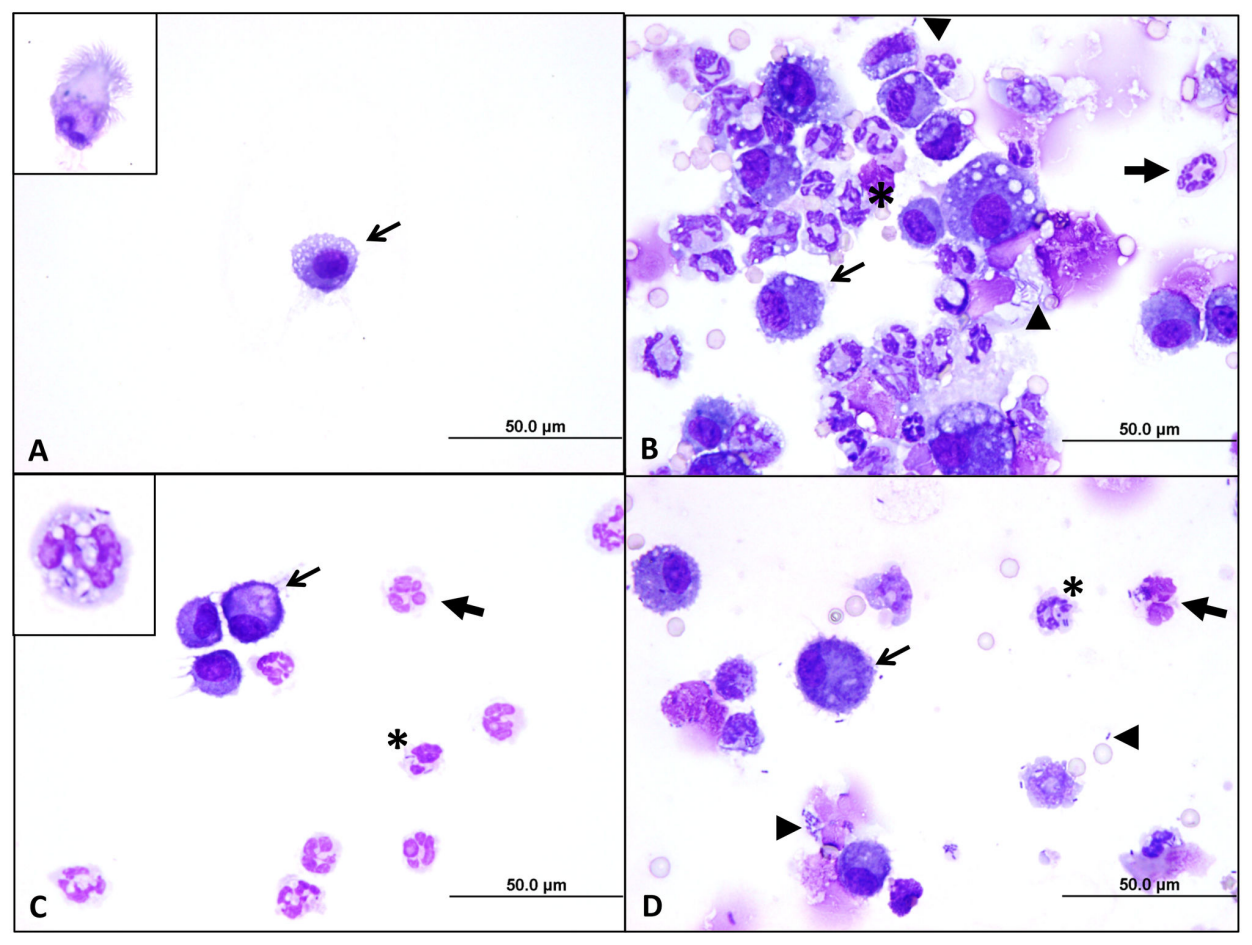
Cytologic evaluation of BALF from infected mice. Bronchoalveolar lavage fluids from control and infected animals (see Figure 5) were concentrated onto a glass slide, fixed with methanol, air-dried, stained with modified Wright-Giemsa, and examined by microscopy (100X objective). Panel A shows a representative field of a sample collected from a mouse inoculated with sterile PBS (control). These control samples are low in cellularity and consist of occasional macrophages (arrow) and ciliated, columnar respiratory epithelial cells (inset). Panel B shows a representative field of a sample from a mouse infected with 10^4^ *B. mallei* bacteria. Large numbers of degenerate neutrophils (block arrows) and foamy macrophages (arrows) are seen admixed with cellular debris and occasional red blood cells. Medium-sized bacilli are seen phagocytosed by neutrophils (asterisk). Extracellular bacteria are also present (arrowheads). Panel C is a representative field of a sample from mouse infected with 10^3^ *B. mallei* cells, and Panel D is a representative field of a sample from mouse infected with 10^3^ *B. pseudomallei* CFU. In both panels, moderate numbers of degenerate neutrophils and foamy macrophages are present, along with intracellular bacilli, illustrating the similar type and magnitude of inflammation elicited by the two species at the same dose. The inset in Panel C demonstrates that neutrophils contain bacilli adhered to their surface and within the lumens of intracytoplasmic vacuoles.

The bronchoalveolar lavage fluids from control mice appeared normal, consisting of occasional clusters of ciliated columnar (respiratory) epithelial cells and rare alveolar macrophages (Figure 6A). No inflammatory infiltrates were observed in these animals. In contrast, BALF from mice infected with *B. mallei* ATCC23344 or *B. pseudomallei* 1026b were inflamed, consisting primarily of degenerate neutrophils (neutrophils that have lost ability to control water homeostasis and have swollen, eosinophilic nuclei) and activated macrophages with few, if any, respiratory epithelial cells. Degenerate neutrophils form in the presence of bacterial endotoxins like LPS, which are known to perforate their nuclear and cellular membranes. For this reason, the presence of degenerate neutrophils in cytologic preparations, even in the absence of bacteria, suggests bacterial infection [65,66]. Neutrophils ingest bacteria by receptor-mediated phagocytosis, a process which internalizes bacteria within a vacuole (e.g. phagosome) with the ultimate goal of fusing that vacuole with a lysosome, where bacteria are digested [67,68]. While the presence of extracellular bacteria and degenerate neutrophils suggests infection, the presence of intracellular bacteria, specifically bacteria contained within intracytoplasmic vacuoles, is indicative of bacterial infection [65,66]. Several neutrophils and macrophages were found to contain phagocytosed bacteria, confirming active infection. The BALF from infected mice also contained cellular debris and free nuclei, which along with the presence of degenerate neutrophils, indicate on-going cell necrosis, presumably due to bacterial endotoxins like LPS. Some lavage samples appeared hemorrhagic, as they possessed low to moderate numbers of erythrocytes (red blood cells [RBCs]) and/or macrophages with intracellular RBCs and hemosiderin pigment (hemoglobin breakdown product from RBCs). Bronchoalveolar lavage fluids from mice infected with 10^4^ organisms possessed the greatest bacterial burden and marked pyogranulomatous inflammation (Figure 6B), while animals inoculated with 10^2^ CFU displayed moderate bacterial numbers and degree of inflammation (data not shown). The BALF from mice infected with 10^3^ organisms demonstrated intermediate inflammation and bacterial burden (Figure 6C and 6D). Overall, no cytologic differences were observed between BALF from mice infected with *B. mallei* ATCC23344 and *B. pseudomallei* 1026b in terms of the type or magnitude of inflammation (when comparing equivalent inoculating doses). Additionally, the presence of hemorrhage and intracellular bacilli in BALF suggests that both organisms may disseminate by both lymphatic and hematogenous routes via infected macrophages or as extracellular bacilli, which is consistent with the results depicted in Figure 4 at the 72-hr time point.

### Selected *Burkholderia* antigens targeted by the mouse immune system

**Figure 7:**
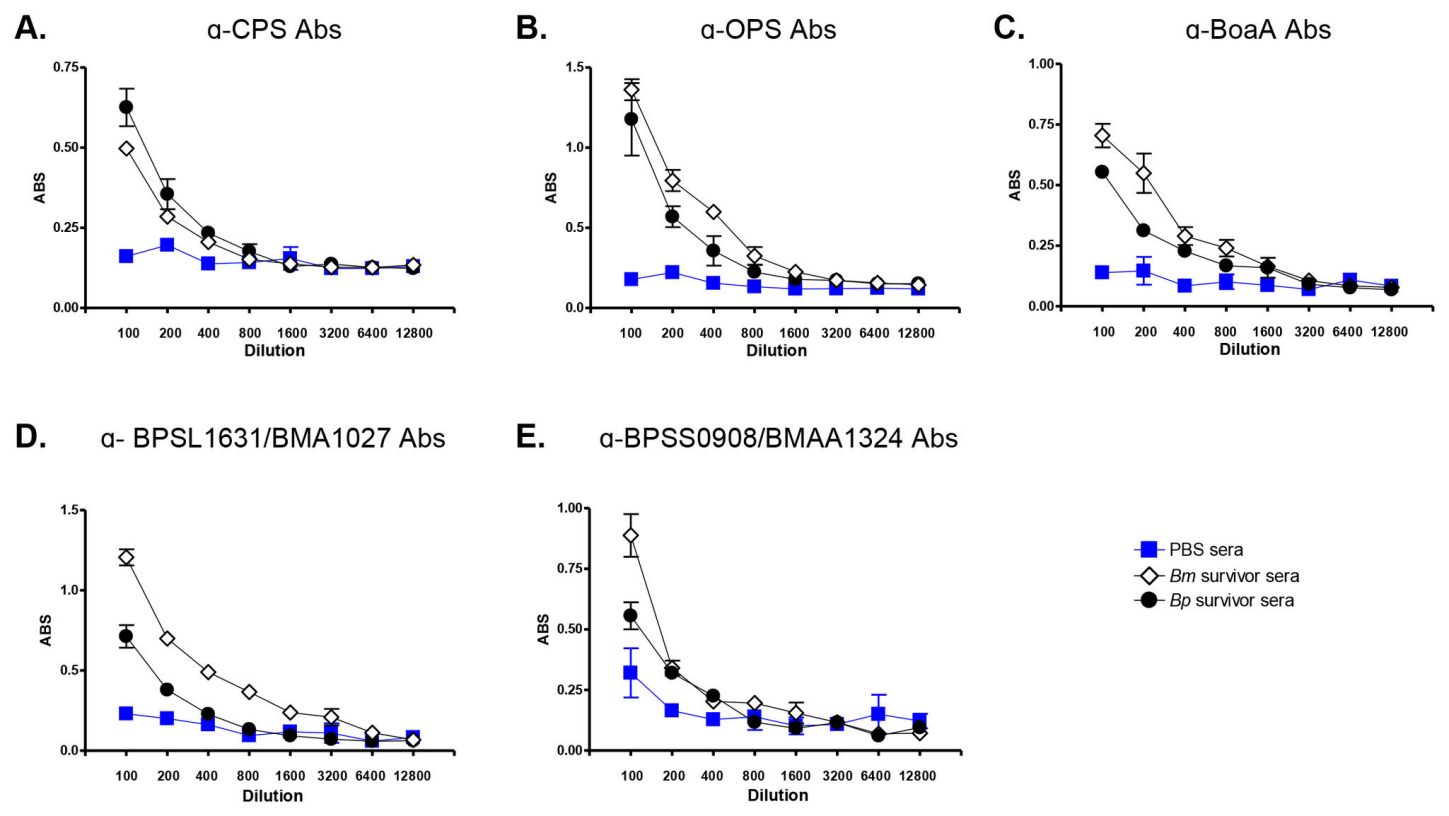
ELISA with sera from mice that survived aerosol challenge with lethal doses of *B. pseudomallei* 1026b (*Bp*) and *B. mallei* ATCC23344 (*Bm*). Serum samples were serially diluted and placed in duplicate wells of plates coated with CPS (panel A), OPS (panel B), His-tagged BoaA (panel C), His-tagged BPSL1631-BMA1027 (panel D), and His-tagged BPSS0908-BMAA1324 (panel E). Goat α-mouse Abs conjugated to HRP were used as secondary Abs. The y-axis shows absorbance at a wavelength of 650 nm, which is indicative of antibody binding to antigens coating the plates. The x-axis represents serial two-fold dilutions of sera starting at 1:100 to 1:12,800. The results are expressed the mean absorbance (± standard error). Open diamonds show sera from mice that survived challenge with *Bm*. Closed circles show sera from mice that survived challenge with *Bp*. Blue squares represent sera from control mice that were inoculated with 50 µL of PBS using the MicroSprayer®.

To gain insight into the immune response to the organisms, we tested survivor sera for the presence of antibodies against a panel of surface-associated *Burkholderia* antigens. Figure 7 shows that infected mice produced antibodies against capsular polysaccharides (CPS, panel A), the oligosaccharide chain of LPS (OPS, panel B), the adhesin BoaA (panel C), and the predicted autotransporter proteins BPSL1631-BMA1027 (panel D) and BPSS0908-BMAA1324 (panel E).

CPS and OPS are the most abundant and immunogenic molecules on the surface of *B. pseudomallei* and *B. mallei* and play critical roles in pathogenesis, as mutants lacking the polysaccharides are severely impaired in virulence [61,69-71]. Both structures are highly conserved throughout these *Burkholderia* species [61,69,72-78] and have been shown to elicit the production of antibodies in human cases of melioidosis and glanders [79-82].

BoaA (ORF BPSS0796-BMAA0649 in the published genomic sequence of *B. pseudomallei* K96243 [58] and *B. mallei* ATCC23344 [54], respectively) is an oligomeric autotransporter adhesin characterized by our laboratory [56] and has been shown to stimulate a strong antibody response during experimental equine glanders [83]. The horse is the natural host and reservoir for *B. mallei* and arguably the most relevant surrogate to study glanders. Sera from horses with experimental glanders were also shown to contain high antibody titers against the predicted autotransporter proteins BPSL1631-BMA1027 and BPSS0908-BMAA1324 [83]. Interestingly, the latter also elicits a robust antibody response in human melioidosis patients [84]. The biological functions of BPSL1631-BMA1027 and BPSS0908-BMAA1324, and their contribution to virulence, have yet to be determined.

Taken together, our data demonstrate that mice infected via the aerosol route with *B. mallei* and *B. pseudomallei* produce antibodies against antigens known to be major targets of the immune response in humans and horses (i.e. natural hosts) during infection. These immunological parallels underscore the usefulness and relevance of our model to study disease by these highly pathogenic organisms and develop countermeasures.

## Conclusion

The use of the MicroSprayer® to inoculate animal species intratracheally and study pulmonary infection has been reported for several agents including *Aspergillus fumigatus* (rats [85]), monkeypox (non-human primates [86]), coronavirus (rats [87]), *Mycobacterium tuberculosis* (mice [88]), and *Chlamydophila pneumoniae* (mice [89]). This study is the first report describing the use of the device to study *B. pseudomallei* and *B. mallei*. Our results demonstrate that this aerosol delivery method produces the hallmarks of melioidosis and glanders (low infectious and lethal doses, rapid replication of agents in the lung and dissemination to spleen, bacteremia) and is consistent with other published models of aerosol infection for the organisms (intranasal, whole-body and nose-only aerosol exposure).

The use of the MicroSprayer® delivery platform offers many advantages. The device uses small volumes and accurately delivers a known number of bacteria directly into the lungs (Figures 1 and 2). The inoculation procedure is also rapid. We routinely infect 80 mice in less than 3 hours, and this time period encompasses anesthesia, inoculation and recovery from anesthesia. Furthermore, the model is safe. All manipulations are performed inside a Biosafety Cabinet, do not involve surgical procedures, and do not require extensive or expensive specialized equipment. These considerations are particularly relevant when studying highly pathogenic organisms under BSL3 containment.

While preparing this manuscript, Revelli and colleagues published a report in which they used a similar intratracheal inoculation approach to study pulmonary melioidosis [90]. These investigators used whole body imaging and histopathology to demonstrate that mice inoculated with *B. pseudomallei* develop the primary focus of infection in the lungs, not the nasal passages. The key difference between their method and our delivery platform is the MicroSprayer®, which produces small aerosol particles. Revelli et al used 22s-gauge needles attached to gastight syringes and infused bacterial liquid suspensions into the trachea [90]. Both intratracheal inoculation approaches provide a route of aerosol infection that circumvents the mouse nasal passageways, reduces the potentially confounding complications due to infection of the nasal associated lymphoid tissues and central nervous system, and thus represent valuable surrogates to study pulmonary disease caused by *B. pseudomallei* and *B. mallei*.
